# Efficacy Evaluation and Tracking of Bone Marrow Stromal Stem Cells in a Rat Model of Renal Ischemia-Reperfusion Injury

**DOI:** 10.1155/2019/9105768

**Published:** 2019-03-20

**Authors:** Ling-Jie Wang, Chang-Ping Yan, Dan Chen, Ting Xu, Sheng He, Hua Zhang, Cheng Xu, Ying Qiao, Zeng-Yu Jiang, Rui-Ping Zhang, Jian-Ding Li

**Affiliations:** ^1^Imaging Department, First Hospital of Shanxi Medical University, Taiyuan, Shanxi Province 030001, China; ^2^Gynecology Department, Affiliated Tumor Hospital of Shanxi Medical University, Taiyuan, Shanxi Province 030013, China; ^3^Imaging Department, Affiliated Shanxi Provincial People's Hospital, Shanxi Medical University, Taiyuan, Shanxi Province 030012, China; ^4^Imaging Department, Affiliated Tumor Hospital of Shanxi Medical University, Taiyuan, Shanxi Province 030013, China

## Abstract

**Objectives:**

The aim of this study was to evaluate the effects of bone marrow stromal stem cells (BMSCs) on renal ischemia-reperfusion injury (RIRI) and dynamically monitor engrafted BMSCs in vivo for the early prediction of their therapeutic effects in a rat model.

**Methods:**

A rat model of RIRI was prepared by clamping the left renal artery for 45 min. One week after renal artery clamping, 2 × 10^6^ superparamagnetic iron oxide- (SPIO-) labeled BMSCs were injected into the renal artery. Next, MR imaging of the kidneys was performed on days 1, 7, 14, and 21 after cell transplantation. On day 21, after transplantation, serum creatinine (Scr) and urea nitrogen (BUN) levels were assessed, and HE staining and TUNEL assay were also performed.

**Results:**

The body weight growth rates in the SPIO-BMSC group were significantly higher than those in the PBS group (P < 0.05), and the Scr and BUN levels were also significantly lower than those in the PBS group (P < 0.05). HE staining showed that the degree of degeneration and vacuole-like changes in the renal tubular epithelial cells in the SPIO-BMSC group was significantly better than that observed in the PBS group. The TUNEL assay showed that the number of apoptotic renal tubular epithelial cells in the SPIO-BMSC group was significantly lower than that in the PBS group. The T2 value of the renal lesion was the highest on day 1 after cell transplantation, and it gradually decreased with time in both the PBS and SPIO-BMSC groups but was always the lowest in the SPIO-BMSC group.

**Conclusion:**

SPIO-labeled BMSC transplantation can significantly promote the recovery of RIRI and noninvasive dynamic monitoring of engrafted cells and can also be performed simultaneously with MRI in vivo for the early prediction of therapeutic effects.

## 1. Introduction

Renal ischemia-reperfusion injury (RIRI) is a common cause of acute renal failure (ARF), which has an increasing incidence and high mortality rates. It also affects early functional recovery and the survival time of transplanted kidneys [1, 2]. The main pathological feature of RIRI is damage to the renal tubules, and thus, the regeneration of renal tubular epithelial cells could contribute to the recovery of renal function. Transplantation of bone marrow stromal stem cells (BMSCs) for the treatment of RIRI is a common research focus, owing to the excellent properties these cells possess, including ease of harvesting, low immunogenicity, multiple differentiation potential, and a decreased number of ethical issues in comparison to the use of other stem cell types. Of particular interest is the fact that BMSCs can differentiate into renal tubular epithelial cells, glomerular cells, and other kidney cells [3]. Moreover, the paracrine properties of engrafted BMSCs are beneficial to the recovery of RIRI [4]. Therefore, to investigate the therapeutic effects of BMSCs for RIRI, we administered BMSCs into a rat RIRI model via the renal artery.

Although serum creatinine (Scr) and blood urea nitrogen (BUN) are key assessment factors of renal function recovery, they cannot reflect the homing and survival of engrafted BMSCs. Dynamic monitoring of the homing and survival of engrafted BMSCs is therefore of great importance in the early prediction of their therapeutic effects.

Magnetic resonance imaging (MRI) is a common and widely used diagnostic technique and is noninvasive and highly sensitive to cell tracking in vivo. Superparamagnetic iron oxide (SPIO) not only can significantly shorten T2 relaxation times to produce dark signals with negative contrast in T2-weighted MRI images, but can also be used to easily label stem cells via endocytosis because of its ultra-small particle size.

The present pilot study was carried out to investigate the effects of SPIO-labeled BMSC transplantation in RIRI and to simultaneously monitor the homing and survival of engrafted BMSCs by MRI for the early prediction of their therapeutic effects. The method may be used as a novel alternative treatment strategy to improve the quality of life of patients with RIRI.

## 2. Methods and Materials

All animal experiments were approved by the Institutional Animal Care and Use Committee of Shanxi Medical University (Approval No, 2016LL141) and complied with the U.S. Guide for the Care and Use of Laboratory Animals 8th Edition 2011 [5]. Eight-week-old female Sprague-Dawley (SD) rats (Animal Center of Shanxi Medical University, Taiyuan, China, SCXK2015-001) were used.

### 2.1. BMSCs Isolation, Culture, Identification, and Pluripotency

After the rats were euthanized via inhalation of an overdose of isoflurane, the tibia and femur were dissected, and the BMSCs were isolated as described previously [6].

The obtained BMSCs were plated in 25-cm^2^ culture flasks (430639; Corning, NY, USA) at a concentration of 1 × 10^9^ cells/L in Growth Medium for Sprague-Dawley Rat MSCs (RASMX-90011; Cyagen, Guangzhou, China) and cultured at 37°C in a 5% CO_2_ incubator (HF90, HealForce, Shanghai, China). After 24 h, nonadherent cells were removed by replacing the culture media. Then, the culture media was changed every 2 or 3 days. When the adherent BMSCs had nearly reached confluence, they were passaged at a density of 6,000/cm^2^. BMSCs from passage 3 were collected and identified using flow cytometry with a Human MSC Analysis Kit (562245; BD, NJ, USA) for expression of CD11b, CD19, CD34, CD44, CD45, CD73, CD90, CD105, and HLA-DR.

To investigate the differentiation character and pluripotency of BMSCs, differentiation was induced in vitro by adding osteogenic and adipogenic culture medium (RASMX-90021 and RASMD-90021; Cyagen, Guangzhou, China), respectively. After successful induction, the differentiated cells were stained with Alizarin Red (G8550; Solarbio, Beijing, China) and Oil Red O (T170516G001; Cyagen, Guangzhou, China), respectively, and observed under a microscope.

### 2.2. Labeling with SPIO and Cell Proliferation Test

Complete culture medium containing 25 *μ*g Fe/mL SPIO (0.5 mmol/mL; Resovist, Schering, Germany) and 350 ng/m” poly-L-lysine (P4832; Sigma, MO, USA) was added to BMSCs and coincubated for 24 h at 37°C in a 5% CO_2_ incubator. Following this, the medium was discarded, and the BMSCs were rinsed three times with phosphate-buffered saline (PBS) to remove unlabeled SPIO.

To identify the SPIO labeling rate of BMSCs, Prussian blue staining was carried out with a Prussian Blue Kit (DJ0003, Leagene, Beijing, China). The intracellular distribution of SPIO nanoparticles was observed using a transmission electron microscope (TEM) (JEM-1011; JEOL, OSA, Japan).

The proliferative capacity of SPIO-labeled BMSCs was assessed by BrdU staining. After incubation for 24 hours in complete culture medium containing 1 *μ*mol/mL BrdU (B8010; Solarbio, Shanghai, China), positive SPIO-labeled BMSCs were labeled with anti-BrdU antibody (AB6326, Abcam, Cambridge, UK). Proliferation curves for SPIO-labeled BMSCs and unlabeled BMSCs were obtained using Cell Counting Kit-8 (CCK-8) (CKO4; Dojindo, Tokyo, Japan) assays. Briefly, SPIO-labeled BMSCs and unlabeled BMSCs were trypsinized and seeded onto 96-well plates (5 × 10^3^ cells/well), respectively. CCK-8 reagent (10 *μ*L) was added into the wells, and the plates were incubated for 2–4 h at 37°C in a 5% CO_2_ incubator, on days 1, 2, 3, 4, 5, and 6, after incubation in the complete culture medium. Following this, the culture medium containing CCK-8 reagent was discarded, and the cells were washed three times with PBS. The absorbance of the cells was measured at 450 nm and recorded using a microplate reader (Infinite M1000; TECAN, CA, USA). All samples were assayed in triplicate.

### 2.3. In Vitro Magnetic Resonance Imaging of SPIO-Labeled BMSCs

SPIO nanoparticles can cause a significant reduction in T2 signal intensity. The SPIO-labeled BMSCs were placed into a 2 mL centrifuge tube at various cells numbers (0, 10^3^, 10^5^, 10^6^, and 2 × 10^6^) and the lower part of the centrifuge tube was filled with a gel. Coronal T2 weighted images were obtained using a 3.0-T clinical MR imager (Magnetom Verio, Siemens, Munich, Germany) with a circular surface coil (diameter 11 cm), and a cell concentration curve was plotted using the T2 values.

### 2.4. RIRI Model Preparation and BMSC Transplantation

After anesthesia was induced in SD rats via inhalation of 2% isoflurane, the abdominal wall was incised obliquely under the left rib arch, fully exposing the left renal hilum. Blood flow of the renal artery was blocked for 45 minutes using a microvascular clamp, after which the clamp was removed. At 1 week after surgery, the RIRI model was validated by hematoxylin-eosin (HE) staining.

Eighteen SD rats were randomly assigned to the following three groups (n = 6/group): (1) the sham-operation group (SO); (2) PBS injection group (PBS); and (3) SPIO-labeled BMSC treatment group (SPIO-BMSC). After the rats underwent abdominal wall incision and renal hilum exposure for 45 minutes, renal artery clamping was performed in the PBS and SPIO-BMSC groups, but not in the SO group. One week after renal artery clamping, 100 *μ*l PBS and 100 *μ*l cell suspension containing 2 × 10^6^ SPIO-labeled BMSCs were injected into the renal artery of rats from the PBS and SPIO-BMSC groups, respectively.

### 2.5. Magnetic Resonance Imaging In Vivo

Rats were anesthetized via intraperitoneal injection of 3.5% chloral hydrate, and then MR imaging of the kidney was performed using a 3.0-T clinical MR imager with a circular surface coil (diameter 11 cm) before RIRI, on day 4 after RIRI, and on days 1, 7, 14, and 21 after cell transplantation. Axial T2*∗* weighted images (T2*∗*WI) and T2*∗* mapping images were obtained using multiple echo sequence. The imaging parameters were as follows: TR, 400 ms; 6 different TE (2.85, 5.45, 8.0, 10.61, 13.15, and 15.76 ms); field of view (FOV), 80 × 80 mm^2^; slice thickness, 2 mm; spacing, 0.5 mm; base resolution matrix, 256 × 256; number of excitation (NEX), 1. Subsequently, 5 regions of interest (ROI) with a diameter of 1 cm in the renal parenchyma at the level of the renal hilum were selected, and T2*∗* values were measured.

### 2.6. Body Weight and Renal Function Assessment

Body weights in each group were measured before IRI and on day 21 after BMSC transplantation, and the body weight growth rate of each group was compared.

On day 21 after BMSC transplantation, 200 *μ*L of blood was collected from the inferior vena cava (IVC) of rats from each group. After centrifugation at 3000 rpm for 5 minutes, Scr and BUN levels were assessed using an automatic biochemical analyzer (AU680; Beckman, CA, USA).

### 2.7. Histological Assessment by HE Staining and TUNEL Assay

On day 21 after BMSC transplantation, the rats were euthanized by inhalation of an overdose of isoflurane, and their kidney tissues were dissected. The obtained kidney tissues were fixed for 48 h in 4% paraformaldehyde, dehydrated using an alcohol gradient, embedded in paraffin, and cut into 5-*μ*m-thick sections. Then, HE staining was performed using an HE kit (G1120; Solarbio, Shanghai, China), and the slices were observed using a phase-contrast microscope (E100, Nikon, Tokyo, Japan).

In addition, a terminal deoxynucleotidyl transferase dUTP nick-end labelling (TUNEL) assay was also performed using an Apoptosis Kit (11772465001; Roche, Basel, Switzerland). Briefly, 5-*μ*m-thick paraffin sections were dewaxed with xylene, hydrated with gradient alcohol, treated for 20 minutes with 0.1% Triton X-100, incubated for 20 minutes with proteinase K, and rinsed three times (5 minutes per wash) with PBS. Then, the TUNEL reaction mixture was added dropwise, reacted for 1 h at 37°C, and rinsed three times (5 minutes per wash) with PBS. Following this, DAPI was added (D9542; Sigma, MO, USA), and the sections were rinsed three times with PBS (5 minutes per wash). Finally, the slices were enveloped with an anti-fluorescence quenching agent and observed under a fluorescence microscope (IX70; Olympus, Tokyo, Japan).

## 3. Results

### 3.1. Morphologic Feature, Cell-Surface Phenotype, and Pluripotent Differentiation of BMSCs

**Figure 1:**
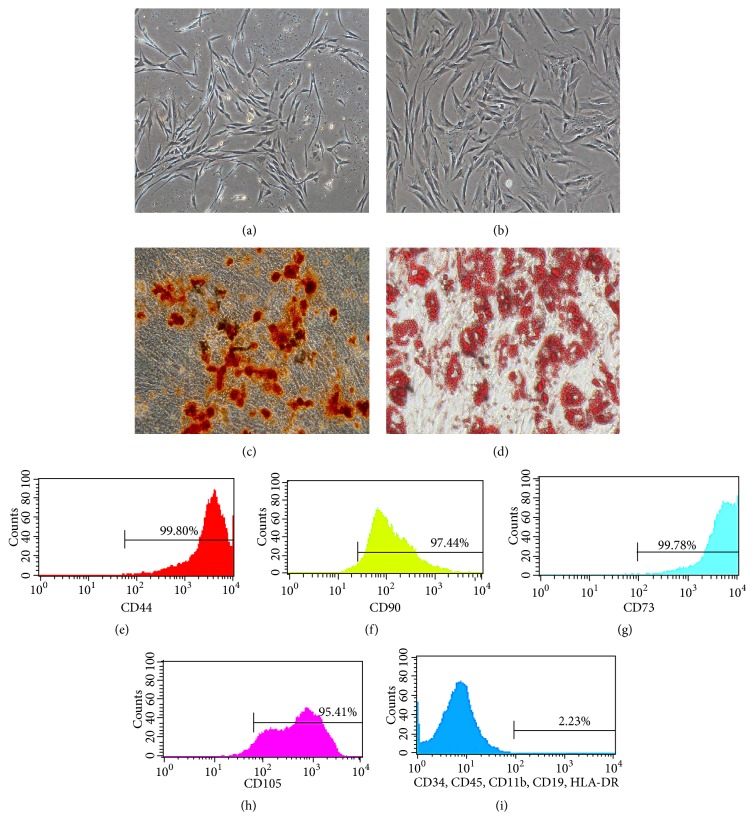
Passage 1 BMSCs on day 7 after isolation (a) and passage 3 BMSCs (b). Magnification, ×40 (a-b). Scale bar, 100 *μ*m (a-b). Alizarin Red staining (c) and Oil Red O staining (d) of BMSCs after induced differentiation of osteogenesis and adipogenesis, respectively. Magnification, ×100 (c-d). Scale bar, 100 *μ*m (c-d). The surface markers of the BMSCs were positive for CD44 (e), CD90 (f), CD73 (g), and CD105 (h) and negative for CD34, CD45, CD11b, CD19, and HLA-DR (i).

Primary BMSCs were isolated using the whole bone marrow adherent method. After 7 days of culturing, the isolated BMSCs grew in clusters, displaying various sizes and spindle shapes, along with many hemocytes scattered amongst them (Figure 1(a)). BMSCs from passage 3 presented as long spindles of the same size and were arranged in parallel (Figure 1(b)).

The cell surface phenotype of the isolated BMSCs from passage 3 was analyzed by flow cytometry, with the results showing that the surface markers of the cells were positive for CD44 (99.80%), CD90 (97.44%), CD73 (99.78%), and CD105 (95.41%) and negative for CD34, CD45, CD11b, CD19, and HLA-DR (a total of 2.25%), consistent with the expression of BMSCs surface markers (Figures 1(e)–1(i)).

The results of Alizarin Red and Oil Red O staining showed that BMSC osteogenesis and adipogenesis could be induced in vitro when cultured with osteogenic and adipogenic media up to 3 weeks, revealing the pluripotency of BMSCs (Figures 1(c) and 1(d)).

### 3.2. Successful Cell Labeling with SPIO, Cytotoxicity Assessment, and Cell Magnetic Resonance Imaging In Vitro

**Figure 2:**
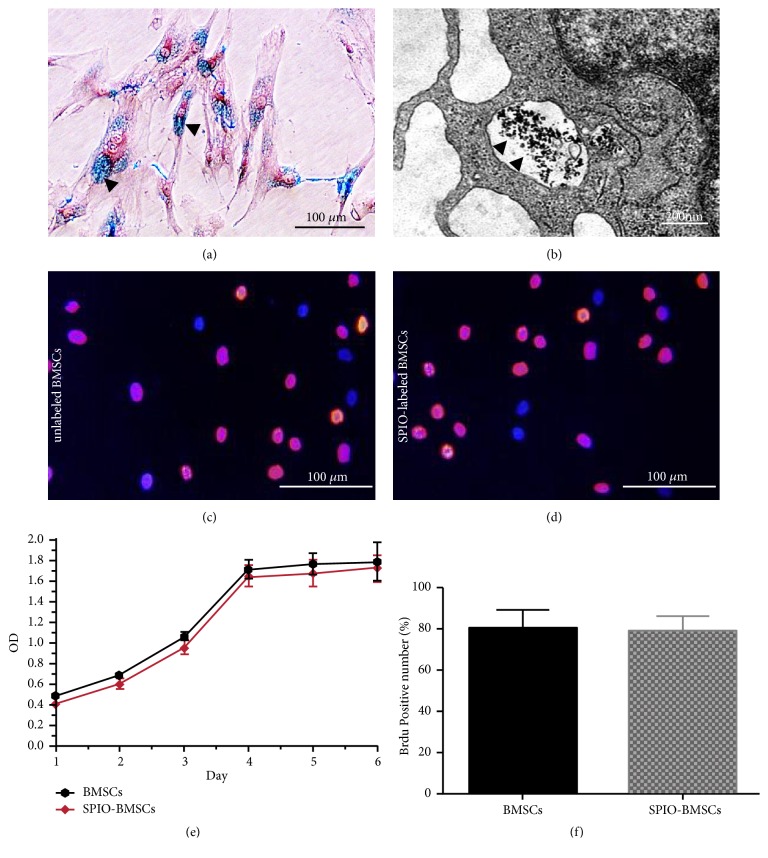
Prussian blue staining showing multiple blue particles (black arrow) in the cytoplasm of SPIO-labeled BMSCs (a). Magnification, ×400. Scale bar, 200 *μ*m. TEM images showed many clusters of black particles (black arrow) in the cytoplasm of SPIO-labeled BMSCs (b). Scale bar, 200 nm. BrdU staining of unlabeled (c) and SPIO-labeled (d) BMSCs. Magnification, ×100 (c-d). Scale bar, 100 *μ*m (c-d). Growth curves of unlabeled and SPIO-labeled BMSCs (e). Bar graph of BrdU-positive cells, with number of unlabeled and SPIO-labeled BMSCs (f).

Prussian blue staining revealed a large number of blue iron particles in the cytoplasm of the SPIO-labeled BMSCs, and the SPIO labeling rate of the BMSCs was nearly 100% (Figure 2(a)). As depicted in TEM images (Figure 2(b)), many clusters of black particles could be observed in the cytoplasm.

There was no significant difference between SPIO-labeled BMSCs and unlabeled BMSCs in terms of positivity for BrdU staining (78.91% ± 7.2% vs. 80.12% ± 9.13%). Cell proliferation curves obtained from the CCK-8 assay also showed that there was no significant difference between the SPIO-labeled BMSCs and unlabeled BMSCs in terms of proliferative ability (Figures 2(c)–2(f)).

These results suggest that SPIO did not show significant cell toxicity at concentrations of 25 *μ*g Fe/ml and was safe for BMSC labeling.

**Figure 3:**
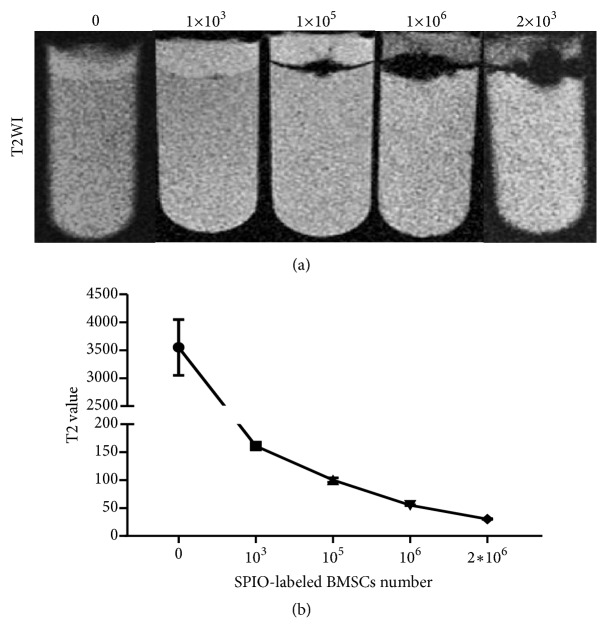
T2-weighted images of SPIO-labeled BMSCs labeled with various different cells numbers in vitro (0, 10^3^, 10^5^, 10^6^, 2 × 10^6^) (a). Curve of T2 value for SPIO-labeled BMSCs changes with cells numbers (b).

T2WI of SPIO-labeled BMSCs at various cell numbers (0, 10^3^, 10^5^, 10^6^, and 2 × 10^6^) showed that the signal intensity of the cell area gradually decreased, with the gray images gradually changing from white to dark black as the number of labeled cells increased (Figure 3(a)). The T2 value cell concentration curve showed that T2 values also gradually decreased (3550.0±499.1 ms, 161.1±5.9 ms, 99.9±4.0 ms, 54.83±0.8 ms, and 30.3±0.5 ms) as the number of labeled cells increased and that the difference was statistically significant (Figure 3(b)).

### 3.3. Successful Preparation of the RIRI Model

**Figure 4:**
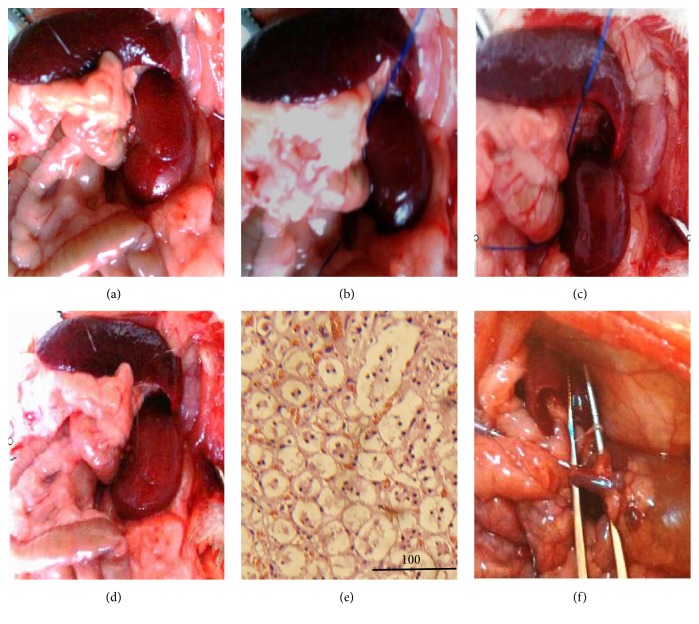
Preparation of an RIRI model and BMSC transplantation. Before the renal artery was clamped (a). Forty-five minutes after the renal artery was clamped (b). 5 minutes (c) and 10 minutes (d) after the clamp was removed. HE staining of kidney tissue at 1 week after RIRI (e). Magnification, ×400. Scale bar, 200 *μ*m. SPIO-labeled BMSCs were transplanted via the renal artery (f).

Before inducing ischemic injury, the color of the left kidney was bright red (Figure 4(a)). After restriction of blood flow for 45 minutes, the color gradually darkened (Figure 4(b)). After the blood flow was restored for 3 minutes, the color became lighter and graniphyric (Figure 4(c)). After a further 10 minutes, the color of the whole kidney became lighter and rosy (Figure 4(d)). One week after ischemia-reperfusion injury, HE staining of the left kidney tissue showed necrosis and partial shedding of renal tubular epithelial cells (Figure 4(e)).

### 3.4. Assessment of the Efficacy of SPIO-BMSCs on Rat RIRI

**Figure 5:**
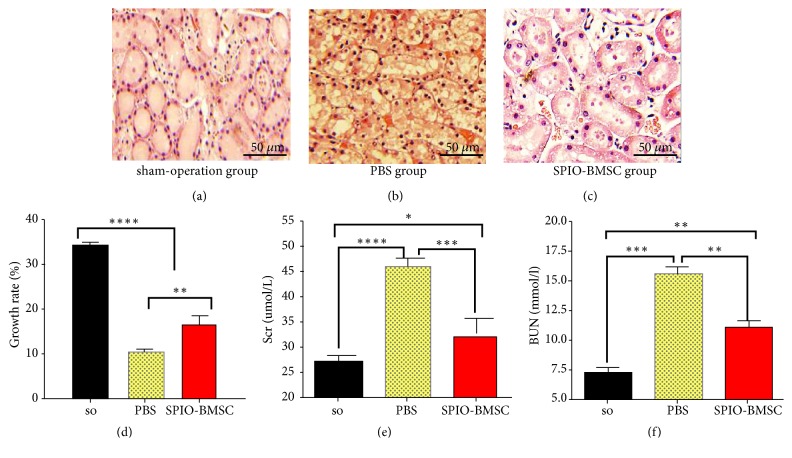
HE staining of kidney tissue on day 21 after cell transplantation in the SO group (a), PBS group (b), and SPIO-BMSC group (c). Magnification, ×100 (A-C). Scale bar, 100 *μ*m (A–C). A bar graph of growth rate (d), Scr (e), and BUN (f) from each group on day 21 after cell transplantation.

The body weight growth rates of the SO, PBS, and SPIO-BMSC groups were 34.15 ± 2.45%, 10.69 ± 1.05%, and 16.83 ± 5.48%, respectively. Renal function test results showed that the Scr levels of the SO, PBS, and SPIO-BMSC groups were 27.12 ± 3.77 *μ*mol/L, 45.83 ± 5.67 *μ*mol/L, and 32.41 ± 3.33 *μ*mol/L, respectively. The BUN levels were 7.23±1.22 mmol/L, 15.58±1.86 mmol /L, and 10.99±2.06 mmol/L, respectively. The body weight growth rate of the SPIO-BMSC group was significantly higher than that of the PBS group (Figure 5(d), P < 0.05), and the Scr and BUN levels were significantly lower than those of the PBS group (Figures 5(e)–5(f), P < 0.05).

On day 21 after cell transplantation, HE staining showed that the renal tubular epithelial cells were arranged regularly and that their structural integrity was sound in the SO group but that they underwent degeneration and necrosis and displayed vacuole-like changes in the PBS group. Although degeneration and vacuole-like changes of the renal tubular epithelial cells were also observed in SPIO-BMSC group, this was to a significantly lower degree than those observed in the PBS group (Figures 5(a)–5(c)).

**Figure 6:**
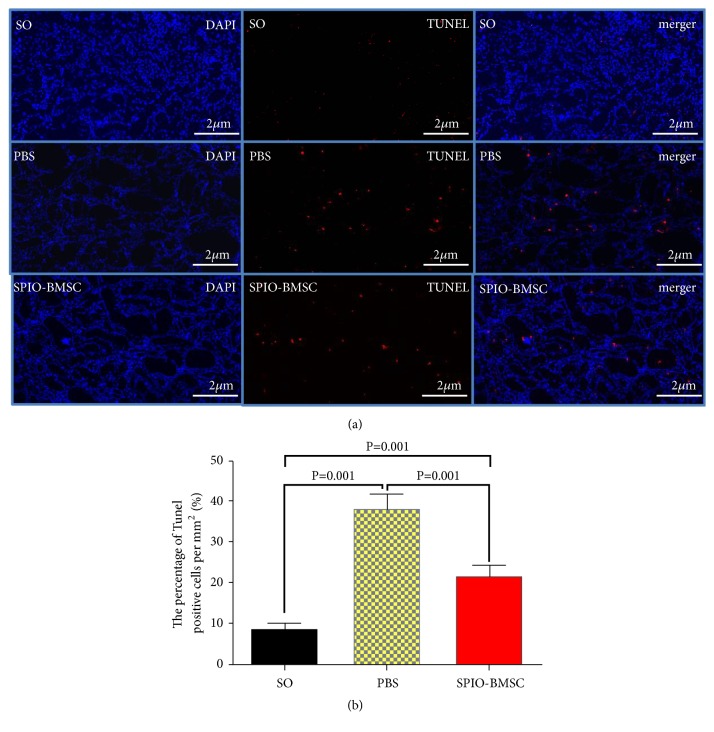
TUNEL staining of kidney tissue on day 21 after cell transplantation in each group (a). Magnification, ×100. Scale bar, 2 *μ*m. A bar graph of the percentage of TUNEL-positive cells per mm^2^ from each group on day 21 after cell transplantation (b), 8.75±1.51% (SO group), 38.33±3.51% (PBS group), and 21.86±2.67% (SPIO-BMSC group).

To assess the apoptosis of renal tubular epithelial cells induced by renal ischemia-reperfusion, a TUNEL assay was performed on day 21 after cell transplantation. As shown in Figure 6, the number of red highlights representing apoptotic renal tubular epithelial cells in the SPIO-BMSC group was significantly lower than those observed in the PBS group.

### 3.5. Tracking SPIO-BMSCs In Vivo by MR

**Figure 7:**
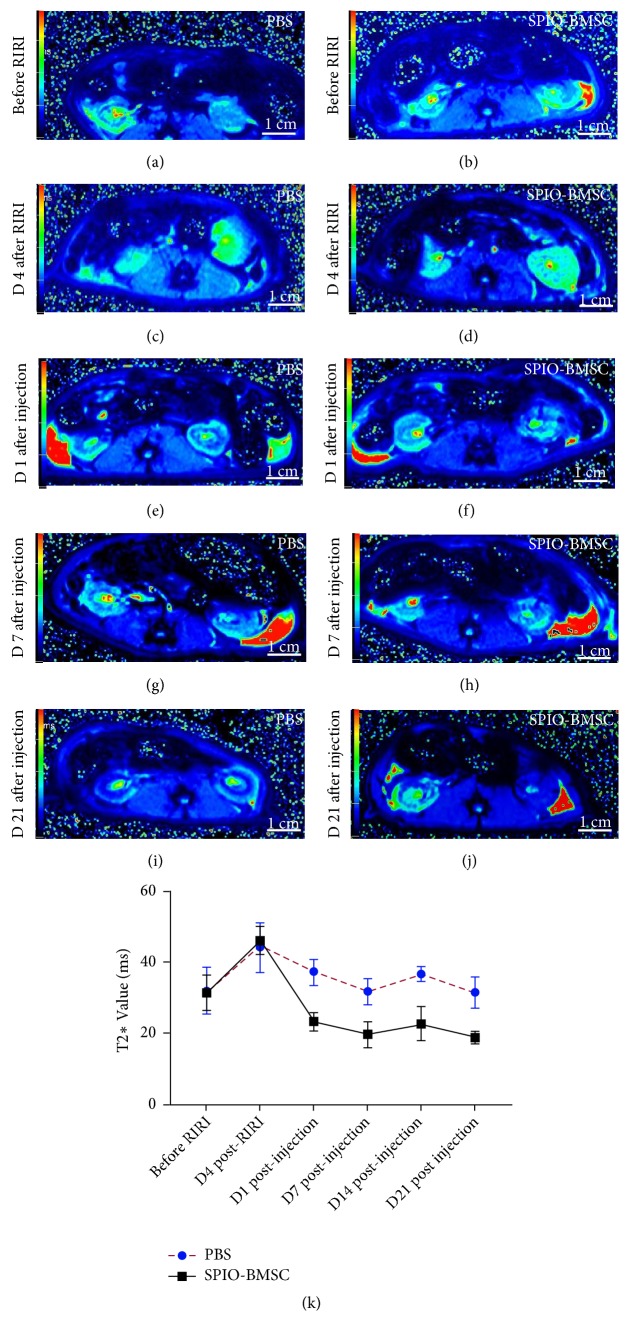
T2*∗*WI of rat kidney in the PBS group before RIRI, on day 4 after RIRI, and on days 1, 7, and 21 after cells transplantation (a, c, e, g, i). T2*∗*WI of rat kidney in SPIO-BMSC group before RIRI, on day 4 after RIRI, and on days 1, 7, and 21 after cells transplantation (b, d, f, h, j). T2*∗* value time curve of the renal lesion in the PBS and SPIO-BMSC groups (k).

Before injury, T2*∗*WI showed the morphology, size, and signal intensity of the kidneys to be roughly symmetrical (Figures 7(a) and 7(b)). After RIRI, the left kidney size and signal intensity increased, and the corticomedullary boundary was unclear in comparison with that of the right kidney.

More low-signal areas in the left renal parenchyma of the SPIO-BMSC group were observed on T2*∗*WI after cell transplantation compared to those in the PBS group (Figures 7(a)–7(j)). The range of these low-signal areas gradually increased over time, which indicated the homing of engrafted BMSCs. Moreover, we observed that the left kidney signal was significantly lower than that of the right on day 21 after cell transplantation. The T2*∗* value time curve of the renal lesion showed that the T2*∗* value decreased gradually with time in the PBS and SPIO-BMSC groups after cell transplantation but that the T2*∗* value of the SPIO-BMSC group was always lower than that of the PBS group after cell transplantation.

## 4. Discussion

RIRI is a potentially devastating pathophysiological development in clinical medicine, is quite often encountered in hospitalized patients, and is a major cause of acute kidney injury (AKI), with a 50% to 80% mortality rate [1, 4, 7, 8]. During kidney transplantation and in certain hypotensive states, subsequent RIRI is also an unavoidable progression, often resulting in severe renal dysfunction [2, 9]. Currently, there are no effective therapeutic strategies for RIRI [10], and treatment still relies on pharmacotherapy. However, pharmacotherapy of RIRI has always yielded an undesirable therapeutic effect. In recent years, stem cell-based technology has proven a powerful and promising treatment for the cure of RIRI, achieving many exciting results. Recently, plentiful data have demonstrated that stem cell-based therapy is beneficial for many acute tissue or organ injuries, such as acute myocardial ischemia, acute liver damage, and acute spinal cord injury [6, 11–17].

Of the wide variety of stem cells available, bone marrow mesenchymal stem cells (BMSCs) are among the least associated with ethical issues. In addition, BMSCs are also a multipotent stem cell population, which are easily harvested, cultured, and used in autologous transplantation protocols. They also have the ability to home in on injured sites and secrete an array of trophic factors and cytokines. It has been demonstrated that BMSCs have the ability to cross lineage boundaries and that they can differentiate into many other types of cells in vivo or in vitro [3, 18]. In the present study, we showed that BMSCs could be induced to differentiate into bone cells and fat cells in vitro. In recent years, the therapeutic effect of BMSC-based against various diseases has been the focus of many investigations. Furthermore, BMSCs have been proven to be effective in promoting nerve regeneration and enhancing neoangiogenesis [19, 20]. Therefore, autologous BMSCs may constitute a very suitable cell source for tissue repair.

Following RIRI, the primary pathologic change is necrosis and apoptosis of tubular cells, which can induce acute kidney injury (AKI) and renal function impairment. Studies have revealed that engrafted BMSCs are capable of actively homing into the lesion area, that they promote the regeneration and proliferation of renal tubular epithelial cells by improving the microenvironment, and that they are able to differentiate into renal tubular epithelial-like cells in vivo after acute renal injury [21–24]. CD44 may play a key role in engrafted BMSCs homing into injured sites [25]. Engrafted BMSCs can significantly improve the microenvironment via IL-10 secretion to ameliorate RIRI [26]. Consequently, autologous BMSC transplantation represents an alternative treatment strategy for RIRI.

In line with these studies, the present study showed that the weight and renal function of rats with RIRI were significantly restored after BMSC injection compared to those of the uninjected BMSC group, demonstrating that BMSC-based treatment is effective against RIRI. In addition, histological results showed lesser tubular epithelial cell necrosis and degeneration in the BMSC transplantation treatment group than those observed in the nontransplanted group after RIRI, which also fully affirmed the effectiveness of BMSC-based treatment.

Safe, early, and accurate detection of RIRI repair is particularly important in the assessment of BMSCs-based treatment effects for RIRI. Although renal biopsy is the gold standard for early assessment of RIRI repair, it has poor repeatability due to its invasiveness [27]. Blood creatinine (Cr) and BUN are always used for detection of renal function, but the changes of these indicators exhibit a time lag in RIRI repair assessment. In addition, glomerular filtration rate (GFR) is regarded as the best indicator for renal function evaluation by renal scintigraphy; however, it is restricted in RIRI repair assessment due to a higher radiation dose of the contrast agents used [28]. It is well known that the homing and survival of engrafted BMSCs play a key role in BMSCs-based treatment for RIRI. Therefore, dynamically monitoring the homing and survival of engrafted BMSCs in vivo can predict the therapeutic effects of BMSCs early.

Cell magnetic labeling is a simple, efficient, and noninvasive method of cells tracing in vivo and has been widely used for stem cell tracing studies of the brain, heart, liver, tendon, and kidney in recent years [29–32]. Because of the fact that they do not display cytotoxicity and provide for long-term effective cellular labeling cells without any transfection agents, SPIO particles have been studied extensively for cell tracking in vivo, producing significant dark signals in T2-weighted images of magnetic resonance imaging (MRI), particularly in T2*∗*-weighted images [32–36]. The present study shows that SPIO-labeled BMSCs not only have good cell viability in vitro, but also can be detected with MRI in kidney lesions up to 21 days after cell transplantation.

## 5. Conclusion

In summary, SPIO nanoparticles can label BMSCs effectively without any transfection agents and have no influence on cell viability. In addition, SPIO will not accumulate in the body because of their short half-life in blood and significantly decrease the signal on T2-weighted images because of a high R2/R1 relaxivity ratio. Therefore, SPIO-labeled BMSC transplantation is an effective alternative treatment strategy for RIRI. At the same time, engrafted cells can be dynamically monitored noninvasively in vivo with MRI, allowing for the early prediction of their therapeutic effects. These results suggest that SPIO-labeled BMSC transplantation may have a wide range of clinical applications.

## Data Availability

All the data used to support the findings of this study are available from the corresponding author upon request.
